# Reflectance Prediction Modelling for Residual-Based Hyperspectral Image Coding

**DOI:** 10.1371/journal.pone.0161212

**Published:** 2016-10-03

**Authors:** Manoranjan Paul, Rui Xiao, Junbin Gao, Terry Bossomaier

**Affiliations:** 1 CM3 Research Unit, School of Computing and Mathematics, Charles Sturt University, Bathurst, NSW, 2795, Australia; 2 Discipline of Business Analytics, the University of Sydney Business School, The University of Sydney, Sydney, NSW, 2006, Australia; National Tsing Hua University, TAIWAN

## Abstract

A *Hyperspectral* (HS) image provides observational powers beyond human vision capability but represents more than 100 times the data compared to a traditional image. To transmit and store the huge volume of an HS image, we argue that a fundamental shift is required from the existing “original pixel intensity”-based coding approaches using traditional image coders (e.g., JPEG2000) to the “residual”-based approaches using a video coder for better compression performance. A modified video coder is required to exploit spatial-spectral redundancy using pixel-level reflectance modelling due to the different characteristics of HS images in their spectral and shape domain of panchromatic imagery compared to traditional videos. In this paper a novel coding framework using *Reflectance Prediction Modelling* (RPM) in the latest video coding standard *High Efficiency Video Coding* (HEVC) for HS images is proposed. An HS image presents a wealth of data where every pixel is considered a vector for different spectral bands. By quantitative comparison and analysis of pixel vector distribution along spectral bands, we conclude that modelling can predict the distribution and correlation of the pixel vectors for different bands. To exploit distribution of the known pixel vector, we estimate a predicted current spectral band from the previous bands using Gaussian mixture-based modelling. The predicted band is used as the additional reference band together with the immediate previous band when we apply the HEVC. Every spectral band of an HS image is treated like it is an individual frame of a video. In this paper, we compare the proposed method with mainstream encoders. The experimental results are fully justified by three types of HS dataset with different wavelength ranges. The proposed method outperforms the existing mainstream HS encoders in terms of rate-distortion performance of HS image compression.

## Introduction

Hyperspectral (HS) images are concerned with the measurement, analysis, and interpretation of spectra acquired from a given scene (or specific object) at a short, medium or long distance. Depending on the different image collecting method, HS imaging systems broadly fall into two categories: airborne and ground-based. [1]. HS images are produced by spectrometers, which divide images into many bands (i.e. wavelength images). This is different from greyscale or colour images (RGB) with only one or a few relatively broad wavelength bands, mostly in three bands (red, green, and blue). HS images can provide a wealth of spectral information, usually beyond visible wavelengths.

A HS image includes hundreds of narrow and contiguous spectral bands. Each band which corresponds to intervals of wavelengths can range from 400*nm* to 2500*nm* or more. A HS image with distinct reflectance-distribution in particular wavelengths [1] shows the distribution of reflectance values in different wavelength bands for a number of different types of objects such as soil, vegetation, and water, which provide a unique signature based on the reflectance values. With the recent development in electronics and imaging sensor design, HS cameras are becoming affordable which opens new applications in agriculture, mineralogy, physics and surveillance around the world in laboratory and industry settings that use HS imaging.

A HS image represents 100 times more data compared to a traditional RGB (red, green, blue) image if we consider the same resolution of a HS image with 300 bands and an RGB image with three color channels. Thus, any processing and transmission of a HS image needs a sophisticated compression strategy. The existing lossless (where original data can be perfectly reconstructed from compressed data) HS image compression standard [2] only provides a very small compression ratio that limits HS applications. Moreover, there is no lossy HS compression standard in existence as yet. We argue that a fundamental shift is required from the existing “original pixel intensity”-based coding approaches using traditional image coders (e.g. JPEG2000) to the “residual” based approaches similar to a video coder (e.g. HEVC [3]) to achieve high compression ratios for HS images. The system can be made analogous to video coding by treating each band as a temporal frame. A high level of correlation exists in bands as they correspond to the same location of objects. However, bands corresponding to different wavelengths have different dynamic ranges, and thus do not lend themselves easily to simple residual coding [4].

Applying a video coder to the HS-residual in a straightforward way would not provide the expected compression due to the larger magnitude of the residual compared to traditional video frames where motion prediction modelling is used. Thus, a significant compression gain could be achieved if we can correctly predict a spectral band from previously encoded bands to minimize the residual in closed loop coding.

In this paper, a novel lossy compression framework using *Reflectance Prediction Modelling* (RPM) for a HS image is proposed. To take advantage of the similarity and variability among contiguous spectral bands, we generate a *common informatics wavelength* (CIW) band in each band using a Gaussian mixture modelling and a linear spectral predicted band by considering the differences of spectral bands. The first wavelength band is encoded as intra-coded and the remainder of wavelength bands in a HS image are coded as inter-coded (similar to video coding) by using two reference bands: one is the immediately previous encoded wavelength band and the other is an *instant* CIW band. Obviously the CIW band is updated before encoding the current band using the spectral predicted band through Gaussian modelling. This is a new approach to using HEVC inter-coding for HS data compression, with RPM noticeably improving the HEVC-inter compression performance in our experiments. The results of the experiment confirm that the proposed compression technique based on RPM outperforms the standalone HEVC and other leading-edge compression techniques in terms of rate-distortion performance.

## Background

Several types of HS image compression studies are available in literature based on spectral decorrelation and spectral dimensionality reduction. One of the commonly used algorithms is based on *Principal Component Analysis* (PCA) [5–7]. PCA is a dimensionality reduction method. The original image is projected onto the subspace based on the variance within the data. It changes the original HS image physical data structure and also costs part of the image data loss. Another approach is to keep HS data in the original spectral dimension. A HS image can be encoded without significant loss of information, for this purpose *optimal compression cube* (OCC) [8] has been introduced. It calculates the average of cross correlation of the intra-band then determines an OCC for higher data redundancy. OCC has well-preserved transform information and can achieve higher rate-distortion performance than PCA-based compression techniques while applying different existing encoder techniques. Tang, Xing, Li and Wang [9] use an adaptive band selection to reduce dimensionality. In this method, adjacent bands are arranged into one group and compressed using the JPEG-LS standard. Other than the above-mentioned methods, PCA-*discrete cosine transform* (DCT) and PCA-JPEG2000 have also been widely applied in HS image compression fields [10–18].

Numerous 2D compression techniques are extended for specific 3D HS data structures, such as 3D *discrete wavelet transform* (DWT) [18–20] and 3D-DCT. In the 2D DCT of the JPEG standard, a block size of 8 × 8 is used for block-based compression. In 3D DCT 8 × 8 × 8, a sub-cube is applied. A number of improved 3D-DCT based approaches are proposed in [21][22]. *Distributed source coding* (DSC) [23–25] schemes have received more attention due to their low complexity and error resilience which satisfy the requirements of onboard compression systems. The *context-based adaptive lossless image codin*g (CALIC) is another commonly used technique for HS image compression [26]. This article [27] extends the CALIC algorithm from 2D to 3D-CALIC.

Other popular compression algorithms for HS images using wavelets are EZW- Embedded Zero-trees *Wavelet* [28][29], the SPIHT (*Set Partitioning in Hierarchical Trees)* [30] and the SPECK (*Set Partitioning Embedded Block)* [31][32]. Modified SPIHT algorithms are also introduced for compressing multispectral images [30]. The SPIHT possesses a number of desirable properties such as competitively good performance, low complexity, and embedded encoding which make it perfectly suited for the task of compressing multispectral images. Moreover, in the majority of cases SPIHT outperforms SPECK [33]. Hence in our experiments, we have chosen to compare our algorithm with state-of-the-art 3D-SPIHT. Additionally, *Wavelet difference reduction* (WDR) is another latterly proposed method for efficient embedded image coding [34][35]. The ASWDR algorithm is a simple modification of the WDR algorithm. Raja and Suruliandi [34] compared WDR and ASWDR in terms of parameters such as PSNR (*peak signal to noise ratio*) and MSE (*mean square error*). According to their results, the ASWDR technique performs better than the WDR.

**Fig 1:**
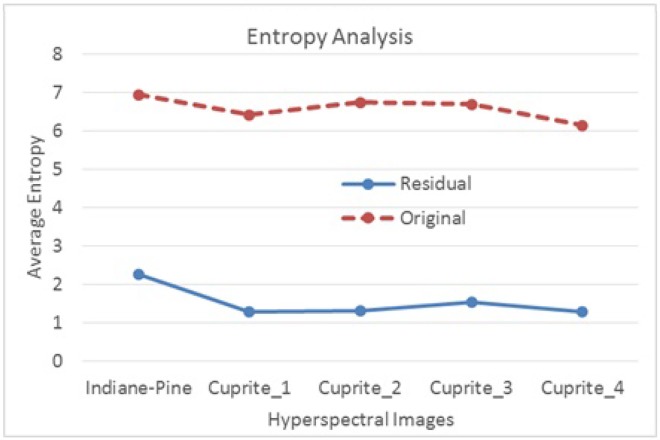
Entropy analysis using original pixel reflectance and residual between adjacent bands for HS images from NASA dataset [10].

Lossless and lossy compression ratios for HS images using the above mentioned original pixel-based encoding techniques are around 3:1 and 80:1, whereas the lossless and lossy compression of a video using the latest video coding standard—i.e. HEVC- are 3:1 and 1000:1 respectively, depending on the application requirements [36][4]. Thus, there is scope to improve the lossy compression ratio with a large margin for a HS image if we fundamentally shift from the existing “original pixel reflectance”-based coding approaches using traditional image coders (e.g. JPEG200) to the “residual”-based approaches using a video coder. Moreover, entropy analysis of HS images, based on the original pixel reflectance and residual between adjacent bands, shows that residual-based entropy is smaller compared to the original pixel reflectance entropy (see Fig 1). Note that smaller entropy provides more compression as it takes fewer bits to encode. The figure reveals that the entropy using residual is only 19% to 33% of the original pixel reflectance for five NASA HS images [10]. The figure indicates that a residual-based encoder—e.g. video coder—should provide better compression compared to the original pixel reflectance-based encoder (i.e. JPEG2000).

The HS image compression technique [37][38] using H.264 (predecessor of HEVC) outperforms a number of conventional image-based HS image compression techniques where both spatial (i.e. intra) and spectral (i.e. inter) correlations are exploited. Although it is a residual-based approach, it could not outperform existing HS compression techniques with large margins due to the relatively larger magnitude of HS-residual between adjacent bands. To achieve better compression we need to develop a predicted band [39] similar to the current band for smaller residual.

Recently *dynamic background modelling* (DBM)-based video coding [40] has attracted more attention due to its capability of better compression and reduction of computational time compared to its rival algorithms. The DBM technique represents background and foreground pixel intensities over time using *Gaussian Mixture* distributions based on means and variances [41]. The DBM exploits uncovered dynamic background areas through a Gaussian model to improve the rate-distortion and computational performance for encoding. The basic idea of DBM is to keep the pixel intensity for background frame generation, which does not change too much over time (i.e. for a number of consecutive frames). HS image structure can be assumed as a video if we consider different wavelength bands as different temporal frames of a video. However, the characteristics of spectral band-images are different compared to video frames. In the video, objects in the foreground are usually more prone to be moving and the background remains somewhat static, so it remains almost the same over multiple frames. In a HS image, spectral reflectance changes over the different bands and there is hardly any object for which reflectance does not change in the spectral bands. Thus, we cannot use a DBM to predict the upcoming wavelength band, as spectral reflectance in each wavelength band is different. Straightforward application of background modelling for HS image compression in the HEVC [3] framework did not provide satisfactory results. Note that a HEVC [42] compression and coding technique should provide comparable compression performance for a HS image compared to other techniques such as JPEG, Wavelet-based encoders, and PCA as it can exploit the residual of the adjacent wavelength bands. This is due to the magnitude of the reflectance-residues between adjacent wavelength bands usually being smaller compared to the original reflectance.

The multiple experiments were performed using three different HS datasets (described in Section III) covering both airborne and ground-based types of HS images to understand the reflectance in different wavelength bands and the performance of different methods including the proposed method. We have compared the proposed HS image coding technique with the relevant state-of-the-art scheme JPEG2000, JPEG, PCA-DCT, HEVC and other wavelet-based encoders 3D-SPIHT, ASWDR and EZW. We observed that the spectral reflectance changes gradually with different bands. Obviously the gradient of changes is varied for different objects. Thus, besides applying DBM in the HS image compression under the HEVC coding framework in a straightforward way, we need to model a CIW band using predicted spectral reflectance so that the generated CIW band is closer to the current wavelength band compared to the previous wavelength band. Using the CIW band as an extra reference band in the HEVC video coding framework should provide more compression while encoding the current wavelength band. This is due to higher similarity with the current band compared to the previously encoded wavelength bands. The results of the experiment confirm that the proposed compression technique based on RPM outperforms the standalone HEVC and other mainstream compression techniques.

## Dataset

### Brimrose HS camera

**Fig 2:**
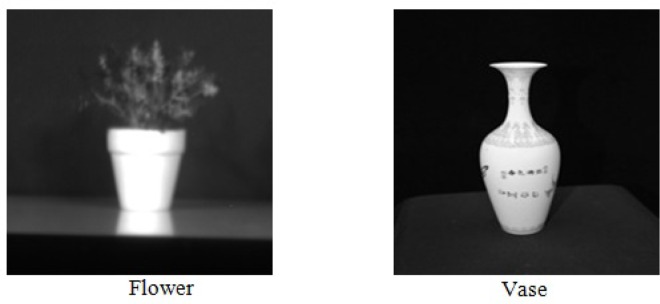
Two examples of Hyperspectral images (for a particular wavelength 500nm) used in the experiments, the images are generated from the raw data provided by Griffith University.

Our experimental HS images were captured using a Brimrose HS camera at Griffith University, Australia, consisting of an array of spectral reflectance of size 1392×1204×61. The images were captured of both indoor and outdoor scenes under daylight illumination. An optical tunable filter was used, capable of acquiring a HS image by sequentially tuning the filter through a series of 61 narrow wavelength bands, each with approximately 10*nm* bandwidth and interval steps at 10*nm*, starting from 400*nm* to 1000*nm*. Two examples of the HS images namely *Pot* and *Vase* are shown in Fig 2. A particular 500*nm* wavelength band in the visual range is used to represent the HS image.

### Natural scenes dataset

On top of the dataset collected from the Brimrose HS camera, another publicly available HS dataset popularly known as natural scenes [7] was also used in the test. The natural scenes dataset consists of a mixture of rural scenes from the Minho region of Portugal, containing rocks, trees, leaves and grass, and urban scenes from the cities of Porto and Braga. Images were obtained during the summers of 2002 and 2003. Particular care was taken to avoid scenes containing movement. Scenes were illuminated by direct sunlight in a clear or almost clear sky. Each HS image has wavelength bands from 400 to 720*nm* with 1018×1339×33 resolutions (however, Scene 5 has 32 bands).

### Airborne visible/infrared imaging spectrometer (AVIRIS)

The dataset used in this part of the experiment is from *Airborne Visible/Infrared Imaging Spectrometer* (AVIRIS) reflectance data [10]. AVIRIS data are mainly collected for identifying, measuring, and monitoring constituents of the earth's surface and atmosphere based on molecular absorption and particle scattering signatures. It delivers calibrated images with 224 contiguous spectral bands and approximately 10*nm* spectral resolutions covering the 0.4–2.5*μm* spectral range. Data was collected from mineral mapping at Cuprite, Nevada.

## Reflectance Prediction Modelling

An RGB image cube has three (Red Green and Blue) colour components, along with wavelength axis. In this aspect a HS image can be considered as an image cube where the wavelength axis is represented by hundreds of contiguous spectral bands. A pixel in a HS image is actually a vector with a dimension equal to the number of spectral bands. Such inter-band spectral information can be used for spectral prediction. In this paper we propose the RPM technique to improve the compression capability of a HS image and Gaussian mixture-based CIW band modelling into the HEVC video-coding framework. Note that we have converted a 16-bit HS image into an 8-bit HS image before applying different encoders.

### Motivation of multispectral data prediction

**Fig 3:**
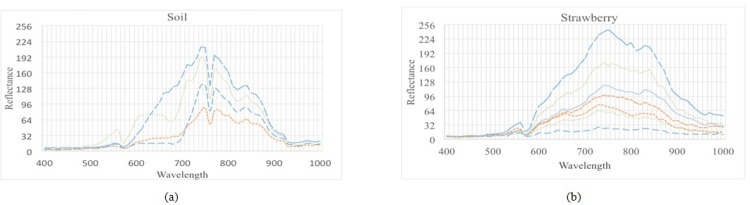
**The distribution of spectral reflectance in different bands: (a) and (b) the effective spectral reflectance of 20 pixels randomly selected in the range of 61 bands (400nm to 1000nm) of two HS images namely ‘Soil’ and ‘Strawberry’ captured by the Brimrose HS camera at Griffith University.**

If we consider spectral information of a HS image, we can easily find the existence of spectral correlation between adjacent bands. The similarity and variability among contiguous bands can be presented preferably by RPM. Fig 3(A) and 3(B) show a graphical representation of the spectral reflectance of randomly selected 20 pixels in 61 wavelength bands ranging from 400*nm* to 1000*nm* using two HS images. Fig 3(C) shows similar reflectance tendency over the different bands using the AVIRIS HS image; the dataset comprises 220 bands with a spectral coverage from 0.4 to 2.5 *μm*. Five pixels representing ‘*soybean’*, ‘*hay*’, ‘*corn’*, ‘*wheat*’ and ‘*wood*’ were selected to plot the reflectance. A given HS matrix for a band can be presented as:

$$HS=\left(\begin{array}{cccc} x_{\left(1,1\right)} & x_{\left(1,2\right)} & \ldots & x_{\left(1,j\right)}\\ x_{\left(2,1\right)} & x_{\left(2,2\right)} & \ldots & x_{\left(2,j\right)}\\ \vdots & \vdots & \ddots & x_{\left(1,1\right)}\\ x_{\left(i,1\right)} & x_{\left(i,2\right)} & \ldots & x_{\left(i,j\right)} \end{array}\right)\in R^{i\times j}$$

Each pixel *x* along spectral bands *n* is a vector. Fig 3A & 3B show different reflectance against different wavelengths for two HS images, namely ‘Soil’ and ‘Strawberry’ captured by Brimrose HS camera at Griffith University. They are mapped into 8-bit reflectance. The reflectance of images with a different range of bands/wavelengths, scene and intensity also demonstrates that probability distribution and correlation of the pixel vector *x* are measureable and predictable. Thus, prediction modelling can be developed through quantitative comparison via spectral similarity measures, allowing for a HS image to present as a single slice key band with spectral shape information.

The observed spectral reflectance in different HS data shows varying degrees of correlation although the rate of increase or decrease might vary depending on the content of the objects in a HS image. The differences in spectral information between two adjacent wavelength bands is typically very small and therefore includes a large amount of data redundancy. For a particular material and a small range of wavelengths the shape of the reflectance/radiance in different bands follow Gaussian distribution. Thus, it is possible to generate a CIW using the existing Gaussian mixture-based modelling [40][41] by customizing the modelling mechanism.

### Customized gaussian mixture modelling for a HS image

A customized DBM based on *Gaussian Mixture Modelling* (GMM) is introduced in this paper. GMM is a weighted sum of *K* Gaussian models to characterize pixels’ intensity of the HS image. When each new slice acquires at the current *n*^*th*^ band, the value of a pixel reflectance is used to match with the GMM models. If a match is found, the pixel intensity is included in the model and updates its parameters based on the newly satisfied pixel intensity. If a match is not found then a new model is introduced where mean is assigned as the new pixel intensity and weight is assigned an arbitrarily high value. If the maximum number of *K* Gaussian models is reached, it is replaced by a new model. Normally the replaced model selected is based on the lowest ratio of weight and standard deviation amongst all models. The model, which provides the highest value of weight and lowest value of standard deviation, is normally classified as the background pixel.

**Fig 4:**
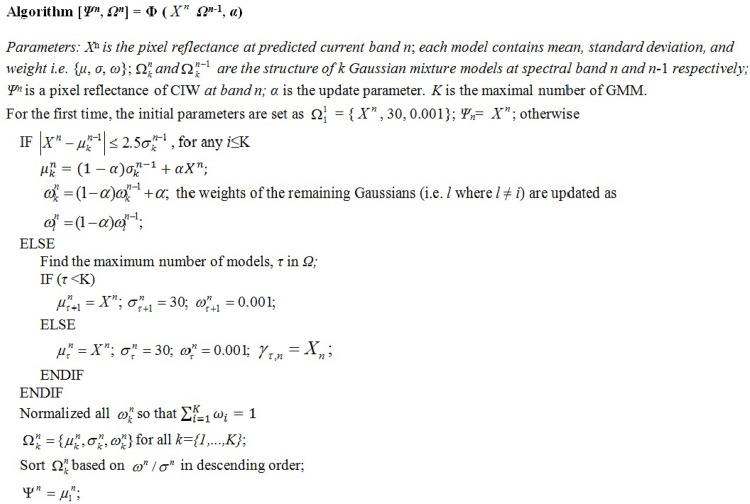
Pseudo code of CIW generation algorithm.

If we assume that the *k*-th Gaussian representing a pixel reflectance with mean *μ*_*k*_, variance $\sigma_{k}^{2}$, and weight *w*_*k*_, then $\sum_{\forall k}\omega_{k}=1$ The Gaussians are always ordered based on *ω*/*σ* in descending order, with the top Gaussian modelling the most stable reflectance. The system starts with an empty set of models and then for every new observation *X*^*n*^ at the current band *n* is first matched against the existing models in order to find one (say the *k*th model) such that $\left|X^{n}-\mu_{k}^{n-1}\right|\leq 2.5\sigma_{k}^{n-1}.$ If such a model exists, its associated parameters, *μ*_*k*_, $\sigma_{k}^{2}$, and *ω*_*k*_, are updated. Otherwise, a new Gaussian is introduced with *μ*_*K*_ = *X*^*n*^, arbitrarily high *σ*, and arbitrarily low *ω* by evicting the last model if it exists (based on *ω*/*σ* in descending order). A pseudo code for the CIW generation steps is given in Fig 4.

1. If the current pixel *X*^*n*^ meets the condition $\left|X^{n}-\mu_{k}^{n-1}\right|\leq 2.5\sigma_{k}^{n-1},$ we consider *X*^*n*^ satisfies with one of the Gaussians models, i.e. a match is found and then updates all relevant parameters.
2. If no match is found with any of the *K* Gaussians; the pixel might be a new background or foreground. Another Gaussian model needs to be introduced with an initial parameter setting [40][41].
3. If the number of the Gaussian models has already reached the maximum number, we need to evict an existing model based on the ratio of weight and standard deviation.

The parameters are updated as follows where *α* is the learning parameter:

$$\mu_{k}^{n}=(1-\alpha )\sigma_{k}^{n-1}+\alpha X^{n};$$ (1)

$$\sigma_{k}^{n^{2}}=(1-\alpha )\sigma_{k}^{{\left(n-1\right)}^{2}}+\alpha {\left(X^{n}-\mu_{k}^{n}\right)}^{T}(X^{n}-\mu_{k}^{n});$$ (2)

$$\omega_{k}^{n}=(1-\alpha )\sigma_{k}^{n-1}+\alpha .$$ (3)

A mixture of *K* Gaussian distribution models is used to model each pixel reflectance independently. The pseudo code to generate a CIW band (i.e. *ψ*) uses pixel reflectance of different bands, which is very similar to the pseudo code described in [39][41]. The main difference is that we use a predicted band pixel reflectance instead of the previously encoded band. The other difference is that we use the mean reflectance value of the maximum weighted model to generate a CIW band rather than the most recent pixel reflectance. A pseudo code of CIW generation is given in Fig 4.

The CIW is an approximation of the current trend of the band based on previously encoded bands. It represents the average trend of the pixel reflectance over different bands. Thus, it is a good approximation of the common informatics of pixel reflectance. The CIW band is very close to the current band, it is used as an extra reference band to encode the current band. Sometimes, the CIW might provide a large residue compared to the current band due to a sudden change of the reflectance of material in a wavelength. To tackle this phenomenon we also use the immediate previous band as another reference band for encoding the current band. The Lagrangian multiplier [43–45] is used to select an appropriate reference image between the CIW and the immediate previous band based on the bit requirements and distortion in block level. Instead of providing each encoded wavelength band into the DBM we use a spectral predicted wavelength band. For example when we are encoding *n*^th^ wavelength band, (*n*-1)^th^ and other previously encoded wavelength bands are used to generate a spectral predicted band, based on the correlation of previous bands, then sending the predicted band to the CIW modelling. Subsequently we use the generated *n*^th^ CIW as an additional reference band to encode the *n*^th^ wavelength band.

### Spectral predictor and compression

**Fig 5:**
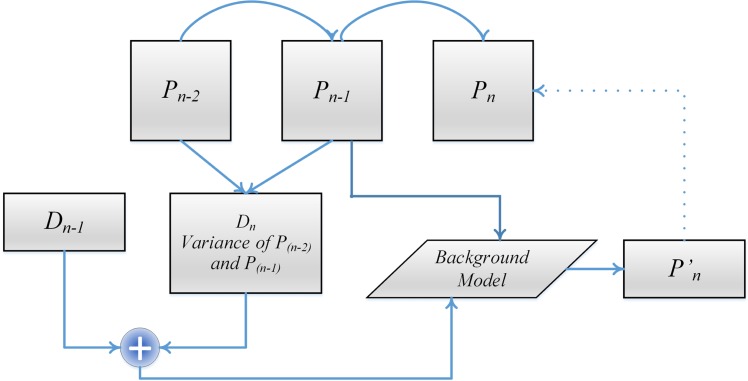
Block diagram of the spectral prediction and referencing technique for the proposed method.

The above-mentioned Gaussian models determine the CIW band using a number of wavelength bands. Our proposed method predicts the current *n*^th^ band by using gradients of two immediate previous encoded bands, i.e. (*n*-1)^th^ and (*n-*2)^th^ bands and previous trend of gradients for creating a reference band. The current gradient is calculated as follows:

$$D_{n}=wD_{n-1}+(P_{n-1}-P_{n-2})(1-w)$$ (4)

where *D*_*n*_ is the weighted gradient for *n*^th^ band using the previous weighted gradient *D*_*n*-1_, the weighted gradient between two recently encoded bands *P*_*n*-1_ and *P*_*n*-2_. We use *w* = 0.3 to balance between the previous gradient and the most recent gradient. To generate a CIW we need to provide a spectral predicted band which is generated by *B*_*n*_ = *P*_*n*−1_ + *D*_*n*_. The rationality of adding the gradient with the previous encoded band to obtain the predicted band is that we assume the variation of the reflectance in the next wavelength follows the same tendency of gradient that it experienced recently. If we assume that the CIW modelling function is Φ, then we can obtain the CIW, $P_{n}^{'}=\Phi (B_{n}).$ $P_{n}^{'}$ and *P*_*n*-1_ are used as reference bands to encode the current *P*_*n*_ wavelength band. The whole prediction process is presented in Fig 5.

## Experiment Result

The RPM algorithm is implemented by using GMM under the HEVC video coding framework. In the proposed method we use dual reference frames where the predicted band is used as the second reference frame and the immediately previous band is used as the first reference frame. In HEVC we use two immediately previous bands as the reference frames. We evaluate RPM with other state-of-the-art image encoders JPEG2000, JPG, HEVC-intra and PCA-DCT at the first set of tests, then by a performance review of RPM with wavelet transform-based encoders, conducted in the next section.

**Fig 6:**
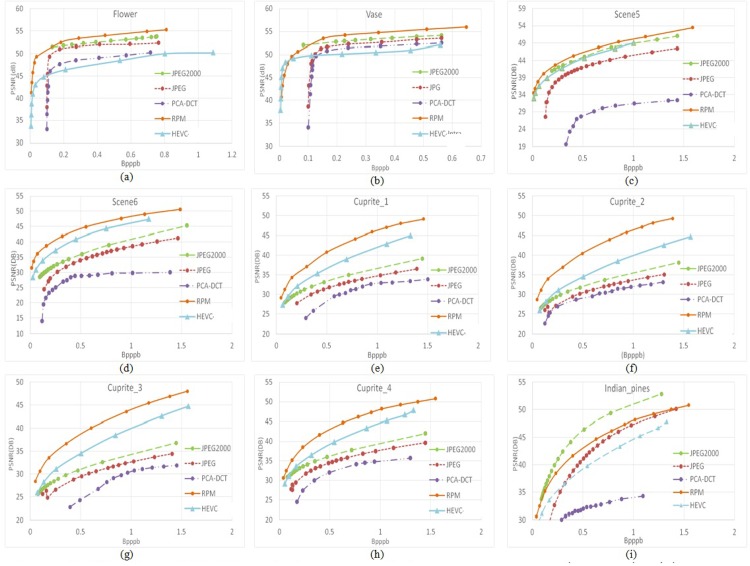
Rate distortion performance of nine HS images using the proposed RPM method, JPEG2000, JPEG, PCA-DCT, and HEVC encoder techniques.

As mentioned in Section III, experiments were performed on test images taken from three sets of images: *Flower* and *Vase*, *Scene5* and *Scene6* and AVIRIS data from Cuprite_1 to Cuprite_4 and Indian_Pine. To evaluate the RPM encoder we present compression performance in Fig 6. It is clear that the proposed RPM scheme outperforms other encoders from the results. The observed difference in PSNR is between 0 to 5.0 dB. Furthermore, JPEG2000 and HEVC are two competitive encoders according to the content of HS sample images. PCA-DCT could not obtain high PSNR because it reconstructs the values of physical bands by eigenvectors. PCA selectively discards part of the energy of a HS image signal since only a subset of the eigenvectors has been selected. It is noteworthy that RPM has the most visible superiority for the Nature Scene and AVIRIS datasets. The reason we reach this conclusion is that the Brimrose HS camera collects *Flower* and *Vase* in the laboratory environment with a small portion of objects and a large portion of black background, where almost all encoders perform in a similar way. The other two datasets Scene and AVIRIS, were collected from complicated outdoor environments, particularly the *Cuprite* set. Consequently, RPM can acquire great prediction results for images of a complicated scene. The performance of the RPM is better compared to other encoders except JPEG2000 for the Indian_Pine HS image. Probable reasons for this would be the different entropy and resolution of the Indian_Pine image compared to the Cuprite HS images. The entropy of the Indian_Pine image based on the residual is 40% larger compared to the Cuprite HS images, and the original resolution of the Indian_Pine HS image is smaller compared to the Cuprite HS images.

As the encoded bands are used in the predicted and CIW generation process in both encoder and decoder, we do not need to encode the CIW band to transmit for the decoder. We use a dual reference band and IPPP format (i.e. the first wavelength band is intra-coded and other bands are predicted as inter-coded) for both standalone HEVC and the proposed RPM algorithm in the HEVC platform. We do not use any motion estimation, as unlike video, each wavelength band has no motion compared to its previous image. However, we use HEVC tree-structured block partitioning to get the best-matched block to minimize residual error.

We know that less prediction error variance always leads to less residual noise, less prediction distortion and higher compression efficiency. Since no scene change happens, CIW-based prediction provides smaller residue compared to the inter-bands’ residue in some cases, and this results in a better compression ratio compared to the HEVC. Moreover, the proposed technique provides better rate-distortion performance compared to some wavelet-based encoders due to its better ability to exploit the correlation between inter-band similarities.

**Fig 7:**
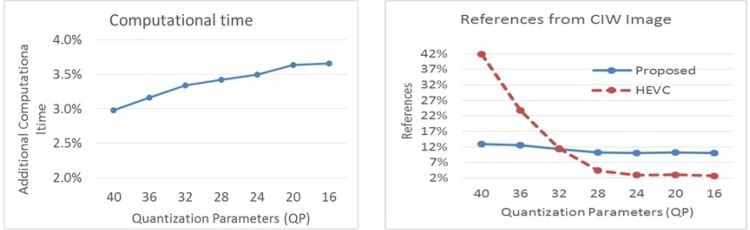
Computational time requirement of the proposed RPM scheme with respect to HEVC (left) and the percentage of reference from the predicted spectral band i.e. CIW band and the 2nd reference band by the proposed scheme and the HEVC respectively.

Fig 7 shows computational time comparison and amount of references used by the second reference band between the proposed RPM scheme and the HEVC scheme. Fig 7 shows that the proposed scheme requires 3~4% extra computational time compared to HEVC, as the proposed scheme needs some computational time for generating the predicted band and the CIW band, through DBM modelling. The figure also reveals that the proposed scheme provides almost constant reference areas using the second reference band, i.e. the CIW band, which is around 10% for different bit rates and quality. On the other hand, the HEVC provides very high reference at the low bit rates and very low reference at high bit rates. This indicates the worthiness of the CIW band as the reference band for a wider range of bit rates.

For the purpose of comprehensive comparisons to a variety of encoders, in this section we have compared RPM with a few standard wavelet based image coders: 3D-SPIHT, ASWDR and EZW. According to the experimental results published in [46], it was demonstrated that 3D-SPIHT can obtain a better compression result; *bit per pixel per band* (bpppb) is lower than 3D-EZW at the same level of PSNR. Therefore, we compare 3D-SPIHT with our method. Furthermore, it is worthy of mention that one of the most recent image compression algorithms is the *adaptively scanned wavelet difference reduction* (ASWDR) algorithm. ASWDR is another benchmark encoder based on wavelets to compare with distortion performance and compression ratio (refer to [34]). The scanning order of ASWDR is different with WDR. It dynamically adapts to the location of edge details in an image and this enhances the resolution of these edges in compressed images. Accordingly, ASWDR exhibits better perceptual qualities, especially at low bit rates.

The rate-distortion results are summarized into two categories, low bit rates (0.5bpppb) and high bit rates (1 bpppb) in Table 1. It can be observed that RPM has presented excellent performance compared to other wavelet-based encoders in most of cases. Particularly outstanding performance is shown in the high bit-rate range, and 3D-SPIHT techniques also provide competitive results.

**Table 1:** Rate distortion performance of four HS images using the proposed RPM method, 3D_SPIHT, EZW, and ASWDR.

Images	Low Bit rates 0.5bpppb (35 PSNR or more)			
	EZW	ASWDR	3D-SPIHT	**RPM**
Cuprite_1	39.39	39.51	39.57	**39.62**
Cuprite_2	35.34	38.41	40.33	**40.45**
Cuprite_3	38.99	39.64	39.85	**40.04**
Cuprite_4	31.36	29.87	31.24	**31.66**
	High bit rate 1 bpppb			
Cuprite_1	45.70	49.13	48.01	**49.23**
Cuprite_2	44.87	43.65	45.98	**47.0**
Cuprite_3	43.93	45.08	44.85	**45.43**
Cuprite_4	42.46	44.72	47.51	**48.19**

**Fig 8:**
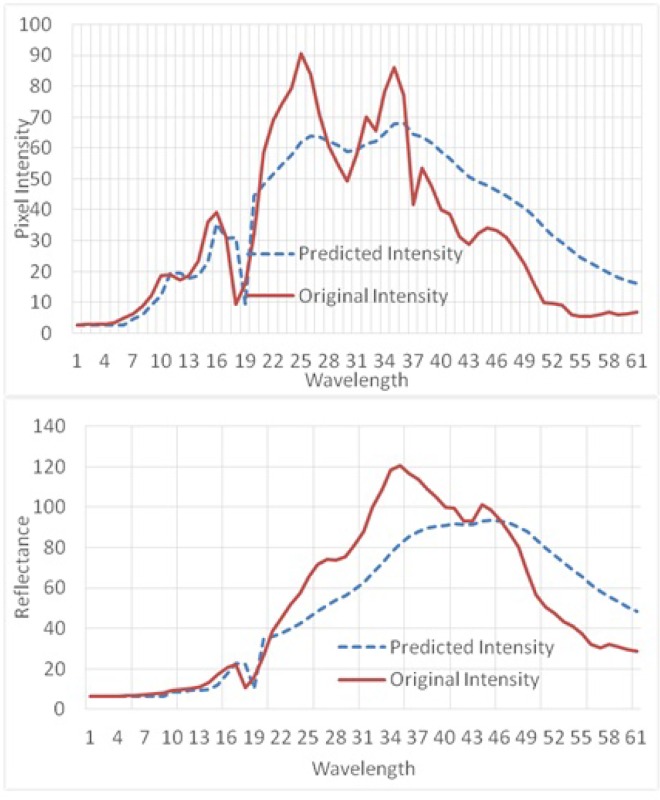
Comparison of predicted reflectance and original reflectance for two sample HS images.

We have conducted some experiments to validate prediction error between a CIW band and the original reference band. For clear comparison we have calculated average pixel reflectance of a randomly selected 16×16 pixel block from the middle of the HS image for both the original bands and the predicted bands. Fig 8 illustrates the comparison results of predicted reflectance and original reflectance for two images. The figure confirms that the predicted pixel reflectance is very close to the original pixel reflectance over the different bands. This indicates that in a number of cases the CIW band is selected against the immediate previous band as a reference band to encode the current band.

## Conclusion

In this paper, HEVC-inter encoding is used for HS data compression under the *Reflectance Prediction Modelling* technique to improve compression capability. We extract an instant spectral band of the HS image using Gaussian mixture-based modelling. The generated instant *common informatics wavelength* is used as the additional reference band for encoding the current band under the latest video coding standard HEVC framework. A HEVC framework is used by considering different bands of a HS image as a different frame or picture of a video.

The proposed HS image coding technique outperforms the relevant state-of-the-art scheme JPEG2000, JPEG, PCA-DCT, HEVC and other wavelet-based encoders 3D-SPIHT, ASWDR and EZW. Overall, the proposed method generates a higher compression rate while maintaining the quality of the image with comparable computational time so that it covers a wide range of applications.
